# Impaired SARS-CoV-2-specific T-cell reactivity in patients with cirrhosis following mRNA COVID-19 vaccination

**DOI:** 10.1016/j.jhepr.2022.100496

**Published:** 2022-04-27

**Authors:** Samer Al-Dury, Johan Waern, Jesper Waldenström, Marko Alavanja, Hevar Hamah Saed, Andreas Törnell, Mohammad Arabpour, Hanna Grauers Wiktorin, Sigrun Einarsdottir, Johan Ringlander, Gisela Ringström, Kristoffer Hellstrand, Anna Martner, Martin Lagging

**Affiliations:** 1 Department of Medicine, Gastroenterology and Hepatology Unit, Sahlgrenska University Hospital, Gothenburg, Sweden; 2 Department of Infectious Diseases, Institute of Biomedicine, Sahlgrenska Academy, University of Gothenburg, Gothenburg, Sweden; 3 TIMM Laboratory, Sahlgrenska Center for Cancer Research, Department of Infectious Diseases, Institute of Biomedicine, Sahlgrenska Academy, University of Gothenburg, Gothenburg, Sweden; 4 Department of Hematology and Coagulation, Institute of Medicine, Sahlgrenska Academy, University of Gothenburg, Sweden; 5 Region Västra Götaland, Sahlgrenska University Hospital, Department of Clinical Microbiology, Gothenburg, Sweden

**Keywords:** Cirrhosis, SARS-CoV-2, COVID-19, vaccination, T cells response, antibody response, BAU, binding antibody units, CAID, cirrhosis-associated immune dysfunction, IFN-γ, interferon-γ, LOD, limit of detection, RBD, receptor-binding domain, S1, spike 1

## Abstract

**Background & Aims:**

Cirrhosis entails elevated risk of COVID-19-associated mortality. This study determined T cell-mediated and antibody reactivity against the spike 1 (S1) protein of SARS-CoV-2 among 48 patients with cirrhosis and 39 healthy controls after mRNA COVID-19 vaccination.

**Methods:**

SARS-CoV-2-specific T-cell reactivity was measured by induced level of T cell-derived interferon-γ (IFN-γ) in blood cells stimulated *ex vivo* with multimeric peptides spanning the N-terminal portion of S1. S1-induced IFN-γ was quantified before and after the 1^st^ and 2^nd^ vaccination (BNT162b2, Pfizer-BioNTech or mRNA-1273, Moderna) alongside serum IgG against the receptor-binding domain (RBD) within S1 (anti-RBD-S1 IgG).

**Results:**

T-cell reactivity against S1 was reduced in patients with cirrhosis after the 1^st^ (*p <*0.001 *vs.* controls) and 2^nd^ (*p <*0.001) vaccination. Sixty-eight percent of patients lacked detectable S1-specific T-cell reactivity after the 1^st^ vaccination *vs.* 19% in controls (odds ratio 0.11, 95% CI 0.03-0.48, *p =* 0.003) and 36% remained devoid of reactivity after the 2^nd^ vaccination *vs.* 6% in controls (odds ratio 0.12, 95% CI 0.03-0.59, *p =* 0.009). T-cell reactivity in cirrhosis remained significantly impaired after correction for potential confounders in multivariable analysis. Advanced cirrhosis (Child-Pugh class B) was associated with absent or lower T-cell responses (*p <*0.05 *vs.* Child-Pugh class A). The deficiency of T-cell reactivity was paralleled by lower levels of anti-RBD-S1 IgG after the 1^st^ (*p <*0.001 *vs.* controls) and 2^nd^ (*p <*0.05) vaccination.

**Conclusions:**

Patients with cirrhosis show deficient T-cell reactivity against SARS-CoV-2 antigens along with diminished levels of anti-RBD-S1 IgG after dual COVID-19 vaccination, highlighting the need for vigilance and additional preventative measures.

**Clinical trial registration:**

EudraCT 2021-000349-42

**Lay summary:**

T cells are a pivotal component in the defence against viruses. We show that patients with cirrhosis have impaired SARS-CoV-2-specific T-cell responses and lower antibody levels after mRNA vaccination against COVID-19 compared with healthy controls. Patients with more advanced liver disease exhibited particularly inferior vaccine responses. These results call for additional preventative measures in these patients.

## Highlights

• After COVID-19 vaccination, patients with cirrhosis had impaired T-cell and antibody responses.
• Child-Pugh class B cirrhosis was associated with poorer immune responses than class A.
• Multivariate analyses excluded potential confounding variables.

## Graphical abstract

**Figure:**
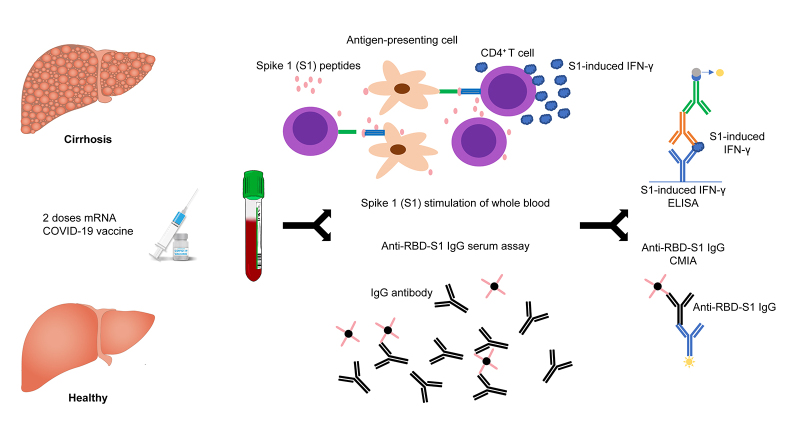

## Introduction

Regardless of aetiology, end-stage liver disease is characterized by impaired immunity. Cirrhosis-associated immune dysfunction (CAID) is believed to arise secondary to injury of hepatic reticuloendothelial cells, reduced hepatic production of proteins crucial for innate immunity^1^ along with systemic inflammation^2^ and translates into a perturbing propensity for severe and life-threatening infections. Although CAID is mostly associated with flawed innate responses,[3], [4], [5] recent studies report that subsets of T cells in patients with cirrhosis express markers of exhaustion, as reflected by expression of TIM-3, CTLA-4, and PD-1, suggesting that T-cell deficiency may contribute to the observed susceptibility to infection.^6^^,^^7^

SARS-CoV-2-infected patients with cirrhosis are at elevated risk of decompensation, severe morbidity, and death.^8^^,^^9^ Thus far scarce data regarding the immunogenicity of COVID-19 vaccines have been reported in these patients. Forty days after immunization with 1 dose of viral vector (Johnson & Johnson) or 2 doses of mRNA (Pfizer-BioNTech or Moderna) COVID-19 vaccines, Thuluvath *et al.* reported that 19% of patients with cirrhosis had suboptimal antibody levels.^10^ Similarly, after 2 doses of viral vector (AstraZeneca) or mRNA (Pfizer-BioNTech or Moderna) vaccines, Ruether *et al.* detected T-cell responses using a cytokine release assay in 17/26 (65%) patients with cirrhosis compared with 19/19 (100%) healthy controls. In the latter study, anti-RBD-S1 IgG levels were similar among patients with cirrhosis and controls.^11^

We aimed to evaluate the immunogenicity of mRNA-based COVID-19 vaccines in patients with cirrhosis by concurrent quantification of T-cell reactivity and anti-S1-RBD IgG. Our results unravel a profound deficiency of T-cell responsiveness against SARS-CoV-2 antigens in cirrhosis paralleled by impaired humoral immunity.

## Patients and methods

### Study population and design

This prospective cohort study was conducted between April and October 2021 at Sahlgrenska University Hospital, Gothenburg, Sweden. Forty-eight patients with cirrhosis of various aetiologies were enrolled among patients attending the outpatient clinic at the Department of Gastroenterology and Hepatology at this hospital (Fig. S1). Patients were diagnosed and examined by a specialist in clinical hepatology. Thirty-nine healthy controls were recruited among healthcare personnel at the Sahlgrenska University hospital as well as their family and friends. The baseline characteristics of patients and controls are detailed in Table 1. Patients or controls with PCR-verified COVID-19 at screening or presence of antibodies against SARS-CoV-2 at initial sampling were not included.

**Table 1:** **Baseline characteristics of the population**.

Characteristics	Patients with cirrhosis	Healthy controls	*p* value
	(n = 48)	(n = 39)	
Age, median, years (range)	63.5 (26–76)	60 (25–86)	
Sex			
Male, n (%)	21 (44)	15 (38)	
Female, n (%)	27 (56)	24 (62)	
Vaccine, Moderna/Pfizer-BioNTech, n (%)	4(8)/44 (92)	2(5)/37(95)	
Days after dose 2 to sampling, median (range)	89 (32–138)	34 (14–147)	<0.001
**Aetiology of cirrhosis**			
Alcohol without other aetiology, n (%)	26 (54)		
NASH without other aetiology, n (%)	6 (13)		
Combined NASH and alcohol, n (%)	4 (8)		
Past hepatitis C without other aetiology, n (%)	1 (2)		
Combined past hepatitis C and alcohol, n (%)	2 (4)		
Autoimmune hepatitis, n (%)	2 (4)		
Cholestatic liver disease (primary biliary cholangitis and primary sclerosing cholangitis), n (%)	4 (8)		
Cryptogenic, n (%)	2 (4)		
**Child-Pugh score**			
Class A, score 5–6, n (%)	31 (65)		
Class B, score 7–9, n (%)	15 (31)		
Class C, score 10–15, n (%)	2 (4)		
**Comorbidities**			
Patient with at least 1 comorbidity, n (%)	37 (77)	8 (21)	
Hypertension, n (%)	17 (35)	4 (10)	
Type 2 diabetes, n (%)	13 (27)		
Osteoporosis, n (%)	6 (13)		
Hypothyroidism, n (%)	6 (13)		
Hyperlipidaemia, n (%)	6 (13)		
Asthma/allergy	5 (10)	3 (8)	
Stroke, n (%)	2 (4)		
**Use of immunosuppressive agents**			
Use of 1 immunosuppressive medication, n (%)	Corticosteroids, 4 (8)	Methotrexate, 2 (5)	
	Azathioprine, 3 (6)		
	Vedolizumab, 1 (2)		
	Total, 5 (10)		
Use of 2 or more suppressive medications, n (%)	Corticosteroids and azathioprine, 3 (6)		

Statistics using Mann-Whitney *U* test. NASH, non-alcoholic steatohepatitis.

The participants received 2 doses of intramuscular mRNA vaccine (BNT162b2, Comirnaty, Pfizer-BioNTech or mRNA-1273, Spikevax, Moderna), at a median 36 (range 26 – 62) day interval. Peripheral blood was collected at baseline, *i.e.* 0-10 days before the 1^st^ vaccination, as well as after the 1^st^ (median 35 days [IQR 25-40 days]) and 2^nd^ (median 89 days [IQR 67-96]) vaccine dose. Serum levels of anti-RBD-S1 IgG and the magnitude of T cell-derived IFN-γ production in response to multimeric spike 1 (S1) peptides after vaccination were predefined primary study endpoints.

### Adverse events

Participants completed a questionnaire regarding adverse events after the 2^nd^ vaccine dose. Adverse events were categorized per the CTCAE (Common Terminology Criteria for Adverse Events) standards.

### Serology

Chemiluminescent microparticle immunoassays were performed on serum using the automated Alinity system for the quantitative measurement of IgG antibodies against the receptor-binding domain (RBD) of the spike protein of SARS-CoV-2 (SARS-CoV-2 IgG II Quant, Abbott, Abbott Park, Illinois, USA) with levels reported in the WHO international standard binding antibody units (BAU)/ml^12^ (quantitative detection range of 14 to 5,680 BAU/ml; samples reaching 5,680 BAU/ml were diluted with seronegative serum and reanalysed allowing for an upper detection limit of >5,680 BAU/ml).

### SARS-CoV-2-specific T-cell reactivity in blood

Vacutainer lithium-heparin tubes (BD, Plymouth, UK) were used to collect peripheral whole blood for assessment of SARS-CoV-2-specific T-cell reactivity. One ml of whole blood was transferred to 10 ml tubes (Sarstedt) and stimulated or not with 1 μg/ml/peptide of 15-mer peptides with 11-amino acid overlap spanning the N-terminal S1 domain of the SARS-CoV-2 S1 (130-127-041, Miltenyi Biotec). After 2 days of incubation at 37°C and 5% CO_2_, the tubes were centrifuged for 5 minutes at 1,500 rpm and plasma was recovered. Plasma was stored at -80°C until analysis of released IFN-γ.

The 15-mer peptides used as stimuli in this assay can be presented on MHC class I and II to activate spike-specific CD8^+^ T cells and CD4^+^ T cells, respectively.

### IFN-γ ELISA

Plasma collected from blood samples with or without S1 peptide stimulation was assessed for interferon-γ (IFN-γ) content by ELISA (DY285B, R&D systems) according to the manufacturer’s instructions. To minimize non-specific reactivity, plasma was diluted (1:2) in PBS containing 1% BSA and 10% mouse serum (Invitrogen). Plates were analysed for optical densities at 450 nm and 570 nm using a FLUOstar Omega plate reader (BMG, Ortenberg, Germany). Levels of IFN-γ induced in response to S1 peptides, with background IFN-γ production in unstimulated samples subtracted, are presented throughout the manuscript. The limit of detection (LOD) of the assay was 10 pg/ml as reported elsewhere,^13^ and thus this threshold was used in the study.

### Acoustic radiation force impulse elastography

Cirrhosis was confirmed at baseline using acoustic radiation force impulse measurement by the ultrasound system Acuson S2000 (Siemens Medical Solutions, Erlangen, Germany). Values were documented in median and interquartile range to median ratio (IQR:median).

### Ethical considerations

All participants gave written informed consent before enrolment. This study was part of the DurIRVac study approved by the Swedish Ethical Review Authority (permit nos. 2021-00539) and by the Swedish Medical Products Agency (Dnr: 5.1-2021-11118). The trial is registered at the European Union Drug Regulating Authorities Clinical Trials Database (EudraCT no. 2021-000349-42).

### Statistical analysis

Continuous variables were described as mean, median, and range of values, as applicable. Categorical data were described with contingency tables including frequency and percent. Mann-Whitney *U* test was applied to calculate differences in serologic/cellular response between groups. The association between various continuous parameters was determined using Spearman’s correlation. Logistic regression was used to calculate the impact of various parameters on cellular and serological immune responses. Parameters with univariate *p* values below 0.1 were included in the multivariate analysis, and the magnitude of response presented as odds ratios with 95% CIs. For some figures, the data were log-transformed, as indicated in the figure text. Values of BAU/ml and pg/ml below the LOD were set to 50% of LOD. Data analyses were performed using SPSS for MacOS and GraphPad Prism 8 for macOS. Statistical significance was set to *p <*0.05. *p* values are designated as follows: ∗*p <*0.05, ∗∗*p <*0.01, and ∗∗∗*p <*0.001. All indicated *p* values are 2-sided.

## Results

### S1-specific T-cell responses after COVID-19 vaccination

**Fig. 1:**
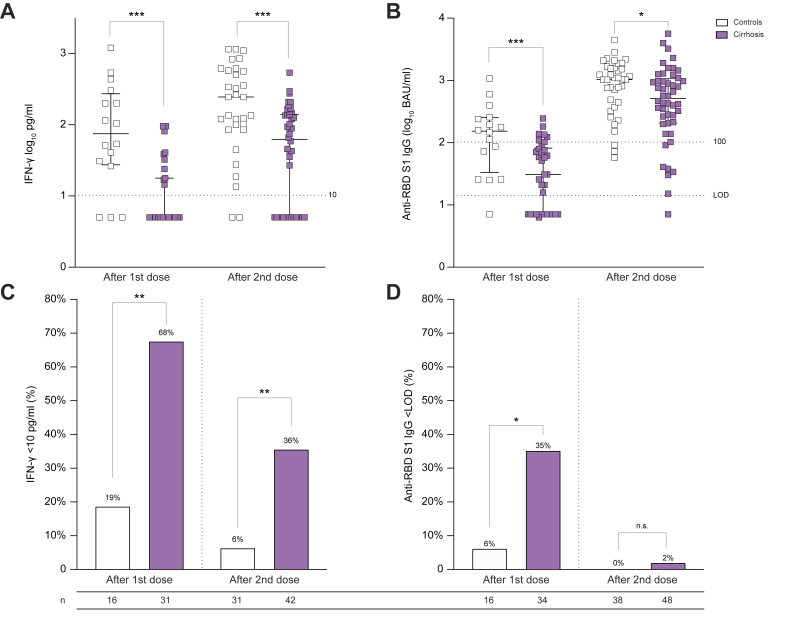
T- and B-cell responses to COVID-19 vaccination in patients with cirrhosis and controls. (A, B) Scatter plots with interquartile range demonstrating IFN-γ in supernatant plasma following stimulation of whole blood with multimeric peptides from the SARS-CoV-2 S1 protein (A), and IgG antibody levels in serum (B) against the RBD within S1 for patients with cirrhosis and healthy controls. The lower dotted line represents the LOD for each assay (S1 IFN-γ <10 pg/ml and anti-RBD S1 IgG <14 BAU/ml). (C, D) Bar charts with percentages of patients with cirrhosis and healthy controls with undetectable IFN-γ (<10 pg/ml) in supernatant plasma following stimulation with S1 peptides (C), and serum anti-RBD-S1 IgG levels below the limit of detection (D). Statistics were calculated by Mann-Whitney *U* test. ∗*p <*0.05, ∗∗*p <*0.01, ∗∗∗*p <*0.001, n.s., not significant. BAU, binding antibody units; LOD, level of detection; RBD, receptor-binding domain; S1, spike 1.

To determine the reactivity of SARS-CoV-2-specific T cells in patients with cirrhosis after COVID-19 vaccination, blood samples collected after the 1^st^ and 2^nd^ vaccine doses were stimulated with multimeric peptides spanning the S1-region of spike 1 followed by analysis of induced levels of T cell-derived IFN-γ. This assay was previously shown to reflect the presence of CD4^+^ and CD8^+^ T cells with specificity for S1-antigens.^13^ The induction of IFN-γ in response to SARS-CoV-2 S1 peptides was impaired in patients with cirrhosis after the 1^st^ (median <10 *vs.* 79 pg/ml in controls, *p <*0.001) and 2^nd^ (median 63 *vs.* 243 pg/ml, *p <*0.001) vaccination (Fig. 1A). Similarly, the proportion of patients with cirrhosis and IFN-γ levels below the LOD (10 pg/ml)^13^ was higher after the 1^st^ (68 % *vs.* 19%, *p <*0.01 *vs.* controls) and 2^nd^ (36% *vs.* 6%, *p <*0.01) vaccination (Fig. 1C).

### Serological responses after vaccination

Similar to the impaired T-cell response, anti-RBD-S1 IgG levels were lower in patients with cirrhosis than in healthy controls after the 1^st^ (median 31 *vs.* 151 BAU/ml, *p <*0.001) and 2^nd^ (median 514 *vs.* 1,044 BAU/ml, *p <*0.05) vaccination (Fig. 1B). Additionally, after the 1^st^ vaccination, a higher proportion of patients with cirrhosis (35%) lacked detectable levels of anti-RBD-S1 compared with controls (6%) (*p <*0.05; Fig. 1D). The characteristics of participants achieving or not achieving detectable cellular immune responses (≥10 pg/ml) and >100 BAU/ml of anti-RBD-S1 IgG after 2 vaccine doses are detailed in Table 2.

**Table 2:** **Characteristics of patients with cirrhosis (n = 48) grouped according to anti-RBD-S1 (<100 *vs.* ≥100 BAU/ml) or interferon-γ (<10 *vs.* ≥10 pg/ml) levels after 2 doses of mRNA COVID-19 vaccine**.

Characteristics	Anti-RBD IgG <100 BAU/ml (n = 7)	Anti-RBD IgG ≥100 BAU/ml (n = 41)	OR (95% CI)	*p* value	Interferon-γ production <10 pg/ml (n = 15)	Interferon-γ production ≥10 pg/ml (n = 27)	OR (95% CI)	*p* value
Median age at vaccination, year (range)	61 (48–73)	64 (26–76)	1.06 (0.95–1.19)	0.27	67 (43–76)	63 (26–71)	0.96 (0.90–1.02)	0.2
Sex female/male, n (%)	5 (71)/2 (29)	22 (54)/19 (46)	0.60 (3.21)	0.55	6 (40)/9 (60)	17 (63)/10 (37)	2.55 (0.7–9.31)	0.16
Child-Pugh class (A-C)	4/3/00	27/12 /2	0.96 (3.84)	0.95	7/8/00	21/4/2	0.50 (0.17–1.49)	0.21
Median ARFI value, kPa (range)	27.2 (14.8–40.2)	22.4 (2.2–56.9)	0.93 (0.84–1.03)	0.17	22.8 (6.2–40.2)	22.5 (5.2–56.9)	1.00 (0.94–1.06)	0.95
Vaccine (Pfizer-BioNTech/Moderna), n (%)	7 (100)/0	37 (90)/4 (10)	1.321	1.002	15 (100)/0	24 (89)/3 (11)	2.781	0.542
Days from vaccine dose 2 to sampling, days, median (range)	90 (41–138)	88 (32–138)	0.98 (0.96–1.01)	0.28	90 (41–109)	88 (14–95)	0.96 (0.92–1.00)	0.07
Immunosuppression, n (%)	0	5 (12)	1.711	1.002	0	5 (19)	4.791	0.142

Statistics using logistic regression. ARFI, acoustic radiation force impulse; BAU, binding antibody units; OR, odds ratio; RBD, receptor-binding domain.

### Impact of Child-Pugh class and concurrent therapy on immune reactivity after vaccination

**Fig. 2:**
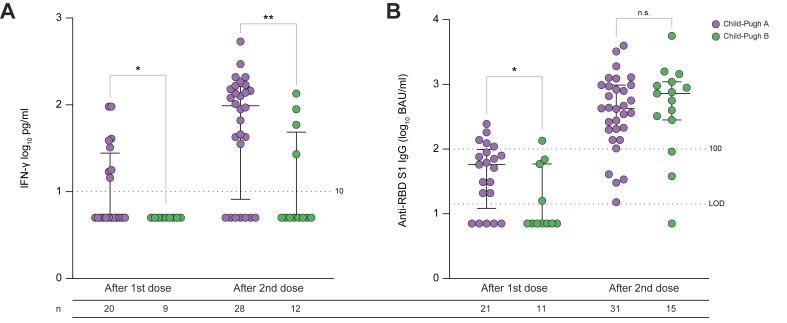
T- and B-cell responses to COVID-19 vaccination in patients stratified by Child-Pugh classification A or B. (A, B) Scatter plots with interquartile range demonstrating IFN-γ in supernatant plasma following stimulation of whole blood with multimeric spike 1 peptides (A) as well as serum anti-RBD-S1 IgG levels (B) in patients with Child-Pugh class A or B cirrhosis. Two patients had decompensated cirrhosis (class C) and were not included in these analyses. The lower dotted line represents the level of detection for each assay (S1 IFN-γ <10 pg/mL and anti-RBD S1 IgG <14 BAU/ml). Statistics were calculated by Mann-Whitney *U* test. ∗*p <*0.05, ∗∗*p <*0.01, n.s., not significant. BAU, binding antibody units; RBD, receptor-binding domain; S1, spike 1.

The Child-Pugh classification (A-C) determines the severity and prognosis of cirrhosis where patients with class A have less pronounced liver disease and more favourable prospects of long-term survival.^14^^,^^15^ Levels of S1-induced IFN-γ were lower in patients with Child-Pugh class B compared with class A after the 1^st^ (*p <*0.05) and 2^nd^ (*p <*0.01) vaccination (Fig. 2A). Similarly, anti-RBD-S1 IgG levels were lower in patients with Child-Pugh class B cirrhosis after the 1^st^ vaccination (*p <*0.05 *vs.* class A; Fig. 2B). The sample size of patients with Child-Pugh class C (n = 2) was insufficient for analysis. No differences were observed regarding T- or B-cell responses among patients with or without ongoing immunosuppressive therapy or with intercurrent disease (Table 2). The aetiology of cirrhosis was diverse and multifactorial, but insufficient sample size prevented meaningful subgroup analyses.

### Multivariable analysis

Logistic regression was performed to determine the impact of potential confounders on the observed differences of vaccine responses. The T cell-derived S1-induced IFN-γ levels were dichotomized based on whether they were above or below 10 pg/ml, which reportedly discriminates infected and uninfected individuals with >95% specificity and sensitivity.^13^ Anti-RBD S1-IgG levels were dichotomized based on whether they were above or below 100 BAU/ml.^16^ The S1-induced IFN-γ response remained significantly inferior in patients with cirrhosis *vs.* controls after the 1^st^ and 2^nd^ vaccination when taking sex, age, vaccine type, intercurrent disease, immunosuppressive therapy, and time from vaccination to sampling into account (Table 3). Similarly, the antibody response after the 1^st^ vaccination remained significantly reduced in patients with cirrhosis in multivariable analysis (Table 4).

**Table 3:** **Logistic regression of S1-interferon-γ production ≥10 pg/ml in the controls and patients with cirrhosis**.

	First vaccine dose				Second vaccine dose			
	OR (95% CI)	Univariate *p* value	Adjusted OR (95% CI)	Adjusted *p* value	OR (95% CI)	Univariate p-value	Adjusted OR (95% CI)	Adjusted *p* value
Cirrhosis	0.11 (0.03–0.48)	**<0.001**	–	–	0.11 (0.02–0.54)	**0.006**	0.09 (0.02–0.57)	**0.01**
Sex (female)	0.65 (0.20–2.12)	0.48	–	–	2.02 (0.69–5.94)	0.2	–	–
Age (years)	0.97 (0.92–1.02)	0.22	–	–	0.97 (0.92–1.03)	0.29	–	**–**
Vaccine (Pfizer-BioNTech)^1^	1.451	0.491	–	–	2.951	0.321	–	–
Immunosuppression	2.22 (0.13–39.6)	0.59	–	–	5.21	0.141	–	–
Time FU-test vaccine 2 (days)	–	–	–	–	0.98 (0.97–1.00)	**0.048**	0.98 (0.96–1.00)	0.06

Statistics using logistic regression. Bold numbers in the tables represent *p* values below 0.05.

FU, follow-up; OR, odds ratio.

1 Fischer’s exact test and likelihood ratio used.

**Table 4:** **Logistic regression of anti-RBD-S1 IgG ≥100 BAU/ml in the controls and patients with cirrhosis**.

	First vaccine dose				Second vaccine dose			
	OR (95% CI)	Univariate *p* value	Adjusted OR (95% CI)	Adjusted *p* value	OR (95% CI)	Univariate *p* value	Adjusted OR (95% CI)	Adjusted *p* value
Cirrhosis	0.16 (0.04–0.58)	**0.005**	–	–	0.50 (0.12–2.09)	0.34	–	–
Sex (female)	0.93 (0.28–3.06)	0.93	–	–	0.92 (0.24–3.52)	0.9	–	–
Age (years)	0.98 (0.93–1.02)	0.33	–	–	0.98 (0.92–1.04)	0.51	–	–
Vaccine (Pfizer-BioNTech)^1^	0.841	1.01	–	–	1.541	1.01	–	–
Immunosuppression	2.08 (0.16–29.96)	0.57	–	–	1.671	1.01	–	–
Time FU-test vaccine 2 (days)			–	–	0.98 (0.96–1.00)	**0.02**	–	–

Statistics using logistic regression. Bold numbers in the tables represent *p* values below 0.05.

BAU, binding antibody units; FU, follow-up; OR, odds ratio.

1 Fischer’s exact test and likelihood ratio used.

### Documented COVID-19 during the study

There were no reported cases of COVID-19 among the participants during the study period (April-October 2021).

### Tolerability and safety

The most commonly reported adverse events were reaction at the injection site (64%) and fatigue (22%). The frequency or severity of adverse events did not differ between patients and controls and no serious adverse events were reported or recorded.

## Discussion

The main finding of this study was that patients with cirrhosis show significantly abated antigen-specific T-cell responses after COVID-19 vaccination. We thus observed that 68% of patients with cirrhosis lacked T-cell reactivity against S1 antigens after the 1^st^ vaccination and that 36% remained non-reactive after the 2^nd^ vaccination. These results confirm and extend those reported by Ruether *et al.* evaluating spike-specific T-cell responses after COVID-19 vaccination.^17^ Multivariable analyses showed that the observed T-cell deficiency was independent of potential confounders, including intercurrent disease or immunosuppressive therapy. However, the limited sample size may have impacted these analyses. We also observed that T-cell dysfunction was significantly more pronounced in Child-Pugh class B cirrhosis than in class A. Thus, 9/9 evaluable patients with class B cirrhosis were completely devoid of T-cell reactivity against S1 antigens after the 1^st^ vaccination, and 8/12 (67%) patients with class B cirrhosis remained non-reactive after the 2^nd^ vaccination. Overall, our findings establish that T cells in patients with cirrhosis respond poorly to SARS-CoV-2 antigens and that the degree of T-cell deficiency is proportional to the severity of liver dysfunction. The serological findings are coherent with those reported by Thuluvath *et al.*,^10^ supporting diminished humoral responses after COVID-19 vaccination in patients with cirrhosis. However, this difference in antibody responses was less pronounced after the second vaccine dose, which may account for the lack of significance noted in some studies.^11^^,^^18^

The immune dysfunction in cirrhosis is primarily associated with flawed innate responses leading to risk of severe and potentially life-threatening bacterial infections.[3], [4], [5] However, T-cell defects including impaired cytokine production elicited by broad T-cell stimulation of blood samples from patients with cirrhosis have been reported.^6^^,^^7^ These findings support that the herein reported T-cell deficiency against SARS-CoV-2 antigens may reflect a generic incapacity to mount T cell-mediated responses to infectious agents in patients with cirrhosis.

Our findings have additional clinical implications including the observation that patients with cirrhosis were largely unprotected after one dose of mRNA vaccine although a catch-up effect regarding antibody responses was noted after the second dose. This is important when considering the waning of immune responses over time after COVID-19 vaccination.^19^^,^^20^ In the USA and many European countries, a third dose of the vaccine has been administered to most patients with chronic liver disease. In Sweden only patients with decompensated cirrhosis thus far have been prioritized, whereas patients with compensated cirrhosis have not been considered a vulnerable population. Our results suggest that additional vaccination be recommended to patients with cirrhosis regardless of whether they have decompensated liver disease.

This study has strengths and limitations. The strengths include the diverse immunological methods and sampling after each vaccine dose along with the possibility of measuring aspects of immunity against SARS-CoV-2 among patients with variable severity of cirrhosis. The limitations include a small sample size and that samples from all patients were not always available for each time-point of analysis, *e.g*. the sample size may have been insufficient to rule out possible associations between vaccine responses and vaccine manufacturer (Pfizer *vs.* Moderna) or immunosupressive therapy. It should be noted that increasing time between the second vaccine dose and subsequent sampling had a weak, albeit significant impact on both diminished humoral and cellular immune responses in univariate but not in multivariate analyses, as demonstrated in Table 3, Table 4, likely reflecting waning immunity. Further studies are required to clarify if the observed T-cell impairment is generic to cirrhosis or to distinct aetiologies of this disease and also if T-cell deficiency impacts on the susceptibility to SARS-CoV-2 infection or on the severity of COVID-19. Also, to define an insufficient humoral response, we used a cut-off anti-RBD-S1 IgG level of 100 BAU/ml, which was previously utilized as a pre-specified marker of poor response in a human therapeutic SARS-CoV-2 vaccine trials^16^ where antibody levels were reported in U/ml equivalent to BAU/ml with no conversion required.^21^ However, this threshold remains to be prospectively validated as a correlate of protection against severe COVID-19 or across variants of SARS-CoV-2.

In conclusion, cirrhosis entails diminished humoral and, in particular, T cell-mediated responses to dual COVID-19 vaccination. Our findings highlight the need for continued vigilance and pre-emptive measures in this vulnerable population.

## Financial support

This work was supported by the 10.13039/501100006310Swedish Medical Research Council (Vetenskapsrådet; grant no. 2021-04779 and 2020-01437), ALF Funds at 10.13039/501100005754Sahlgrenska University Hospital (ALFGBG-438371), the 10.13039/501100007687Swedish Society of Medicine (SLS-961779 and SLS-961159) and the Gothenburg Society of Medicine (GLS-961772).

## Authors’ contributions

SAD, JWae and ML were responsible for designing and writing the protocol, conducting the study, extracting, and analysing data, interpreting results, writing the manuscript, updating reference lists, and creating the table and figure. JWal, AM and KH were responsible for designing and writing the protocol, extracting, and analysing data, interpreting results, writing the manuscript, updating reference lists, and creating the table and figure. MAl, HHS, SE, JR, and GR participated in interpreting results and writing the manuscript. AT, MAr, and HGW were responsible for performed the T cell as well as participated in extracting and analysing data and interpreting results.

## Data availability statement

For original data, please contact martin.lagging@medfak.gu.se. As per Swedish law, individual participant data will not be shared.

## Conflict of interest

The authors declare no conflicts of interest that pertain to this work.

Please refer to the accompanying ICMJE disclosure forms for further details.

## References

[1] Albillos A., Lario M., Alvarez-Mon M. (2014). Cirrhosis-associated immune dysfunction: distinctive features and clinical relevance. J Hepatol.

[2] Albillos A., Martin-Mateos R., Van der Merwe S., Wiest R., Jalan R., Alvarez-Mon M. (2021). Cirrhosis-associated immune dysfunction. Nat Rev Gastroenterol Hepatol.

[3] Irvine K.M., Ratnasekera I., Powell E.E., Hume D.A. (2019). Causes and consequences of innate immune dysfunction in cirrhosis. Front Immunol.

[4] Tuchendler E., Tuchendler P.K., Madej G. (2018). Immunodeficiency caused by cirrhosis. Clin Exp Hepatol.

[5] Noor M.T., Manoria P. (2017). Immune dysfunction in cirrhosis. J Clin Transl Hepatol.

[6] Lebosse F., Gudd C., Tunc E., Singanayagam A., Nathwani R., Triantafyllou E. (2019). CD8(+)T cells from patients with cirrhosis display a phenotype that may contribute to cirrhosis-associated immune dysfunction. EBioMedicine.

[7] Rueschenbaum S., Ciesek S., Queck A., Widera M., Schwarzkopf K., Brune B. (2020). Dysregulated adaptive immunity is an early event in liver cirrhosis preceding acute-on-chronic liver failure. Front Immunol.

[8] Choudhary N.S., Dhampalwar S., Saraf N., Soin A.S. (2021). Outcomes of COVID-19 in patients with cirrhosis or liver transplantation. J Clin Exp Hepatol.

[9] Bajaj J.S., Garcia-Tsao G., Biggins S.W., Kamath P.S., Wong F., McGeorge S. (2021). Comparison of mortality risk in patients with cirrhosis and COVID-19 compared with patients with cirrhosis alone and COVID-19 alone: multicentre matched cohort. Gut.

[10] Thuluvath P.J., Robarts P., Chauhan M. (2021). Analysis of antibody responses after COVID-19 vaccination in liver transplant recipients and those with chronic liver diseases. J Hepatol.

[11] Ruether D.F., Schaub G.M., Duengelhoef P.M., Haag F., Brehm T.T., Fathi A. (2022). SARS-CoV2-specific humoral and T-cell immune response after second vaccination in liver cirrhosis and transplant patients. Clin Gastroenterol Hepatol.

[12] Kristiansen P.A., Page M., Bernasconi V., Mattiuzzo G., Dull P., Makar K. (2021). WHO International Standard for anti-SARS-CoV-2 immunoglobulin. Lancet.

[13] Törnell A., Grauers Wiktorin H., Ringlander J., Arabpour M., Nilsson M.R., Nilsson S. (2022 Jan 12). Rapid cytokine release assay for analysis of SARS-CoV-2-specific T cells in whole blood. J Infect Dis.

[14] Durand F., Valla D. (2005). Assessment of the prognosis of cirrhosis: Child-Pugh versus MELD. J Hepatol.

[15] Albers I., Hartmann H., Bircher J., Creutzfeldt W. (1989). Superiority of the Child-Pugh classification to quantitative liver function tests for assessing prognosis of cirrhosis. Scand J Gastroenterol.

[16] Hall V.G., Ferreira V.H., Ku T., Ierullo M., Majchrzak-Kita B., Chaparro C. (2021). Randomized trial of a third dose of mRNA-1273 vaccine in transplant recipients. N Engl J Med.

[17] Ruether D.F., Schaub G.M., Duengelhoef P.M., Haag F., Brehm T.T., Fathi A. (2021). SARS-CoV2-specific humoral and T-cell immune response after second vaccination in liver cirrhosis and transplant patients. Clin Gastroenterol Hepatol.

[18] Bakasis A.D., Bitzogli K., Mouziouras D., Pouliakis A., Roumpoutsou M., Goules A.V. (2022). Antibody responses after SARS-CoV-2 vaccination in patients with liver diseases. Viruses.

[19] Science Brief (2020).

[20] Levin E.G., Lustig Y., Cohen C., Fluss R., Indenbaum V., Amit S. (2021). Waning immune humoral response to BNT162b2 covid-19 vaccine over 6 months. N Engl J Med.

[21] Jochum S., Kirste I., Hortsch S., Grunert V.P., Legault H., Eichenlaub U. (2021). Clinical utility of Elecsys Anti-SARS-CoV-2 S assay in COVID-19 vaccination: an exploratory analysis of the mRNA-1273 phase 1 trial. medRxiv.

