# Supplementary material for: Impaired SARS-CoV-2-specific T-cell reactivity in patients with cirrhosis following mRNA COVID-19 vaccination
Source: JHEP Rep. 2022 Apr 27;4(7):100496. doi: 10.1016/j.jhepr.2022.100496 (PMC9045869; doi:10.1016/j.jhepr.2022.100496)

Impaired SARS-CoV-2-specific T-cell reactivity in patients with cirrhosis following mRNA COVID-19 vaccination

Samer Al-Dury, Johan Waern, Jesper Waldenström, Marko Alavanja, Hevar Hamah Saed, Andreas Törnell, Mohammad Arabpour, Hanna Grauers Wiktorin, Sigrun Einarsdottir, Johan Ringlander, Gisela Ringström, Kristoffer Hellstrand, Anna Martner, Martin Lagging

Table of content

Fig. S1..2

Fig. S1. Flowchart of the study cohort.

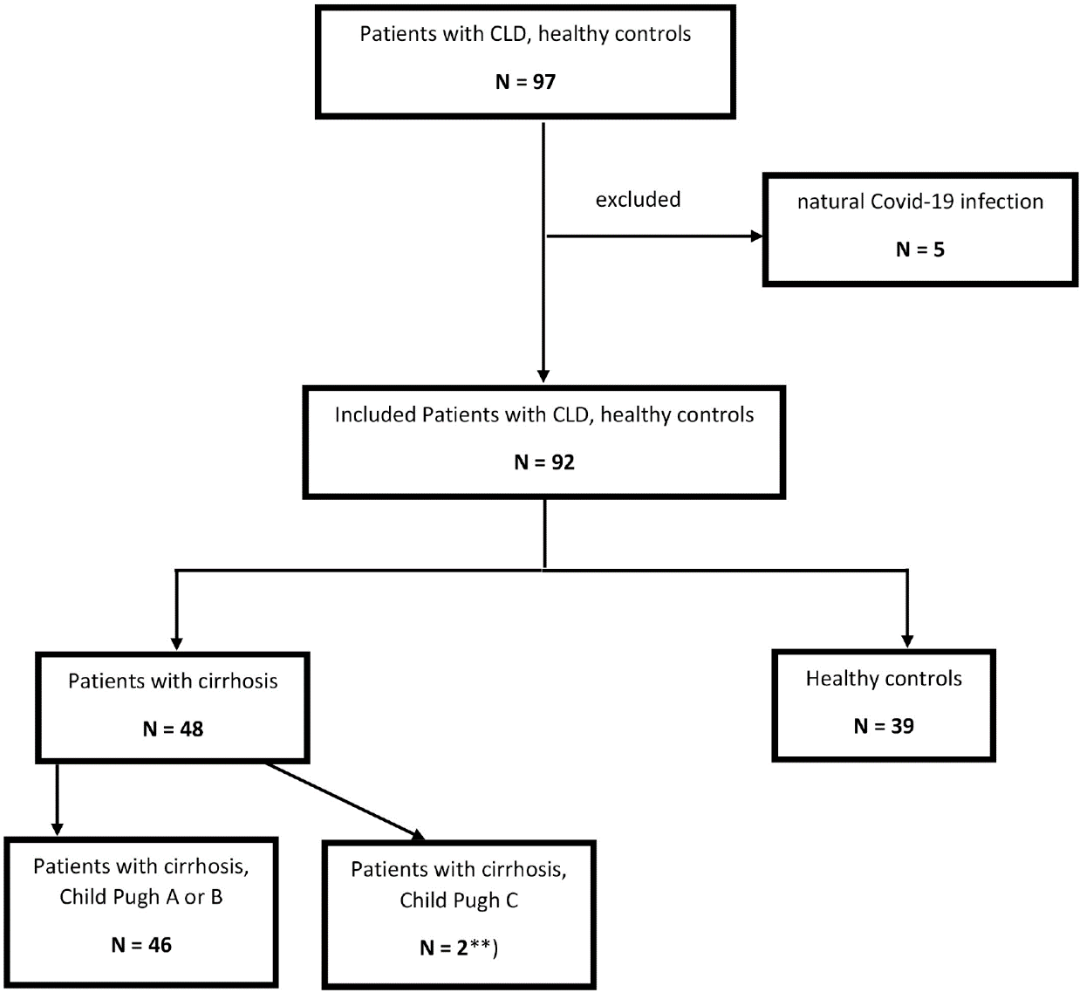

Supplement: Multimedia component 1 [file mmc1.pdf]
