# Supplementary material for: Impaired SARS-CoV-2-specific T-cell reactivity in patients with cirrhosis following mRNA COVID-19 vaccination
Source: JHEP Rep. 2022 Apr 27;4(7):100496. doi: 10.1016/j.jhepr.2022.100496 (PMC9045869; doi:10.1016/j.jhepr.2022.100496)
Supplement: Multimedia component 2 [file mmc2.pdf]

admincontrol

2021-00539 Grundansokan godkand av ordforande.pdf

Signers:

Name	*Method*	*Date*
Bengt Magnus Forsberg	BANKID	2021-03-26 13:33 GMT+1

This document package contains:

- Front page (this page)
- The original document(s)
- The electronic signatures. These are not visible in the document, but are electronically integrated.

This file is sealed with a digital signature. The seal is a guarantee for the authenticity of the document.

Document ID: 1CD6ABBE44414D4F9565A33BAB10AC6D

THE SIGNED DOCUMENT FOLLOWS ON THE NEXT PAGE >

Dnr 2021-00539
EudraCT-nr 2021-000349-42
Umeå avdelning medicin

BESLUT

Sökande forskningshuvudman
Västra Götalandsregionen

Forskare som genomför projektet
Martin Lagging

Projekttitel
Utvärdering av tester för att fastställa hållbarheten hos immunsvar mot SARS-CoV-2 efter naturlig infektion och vaccination

---

Etikprövningsmyndigheten beslutar enligt nedan.

BESLUT

Etikprövningsmyndigheten godkänner den forskning som anges i ansökan.

På Etikprövningsmyndighetens vägnar

Magnus Forsberg
Ordförande

Beslutet har fattats efter föredragning av vetenskaplig sekreterare Thomas Brännströn.

Beslutet sänds till
Ansvarig forskare: Martin Lagging
Forskningshuvudmannens företrädare: Peter Brodin

This file is sealed with a digital signature. The seal is a guarantee for the authenticity of the document.
Document ID:
1CD6ABBE44414D4F9565A33BAB10AC6D
