# Supplementary material for: Impaired SARS-CoV-2-specific T-cell reactivity in patients with cirrhosis following mRNA COVID-19 vaccination
Source: JHEP Rep. 2022 Apr 27;4(7):100496. doi: 10.1016/j.jhepr.2022.100496 (PMC9045869; doi:10.1016/j.jhepr.2022.100496)
Supplement: Multimedia component 3 [file mmc3.docx]

CLINICAL STUDY PROTOCOL

| **Evaluation of the Durability of Immune Responses to SARS-CoV-2 Using Novel Rapid Spike Interferon-Gamma Release and Activation-Induced Marker Assays and Anti-Spike IgG Following COVID-19 Vaccination**  (**DurIRVac**) | |
| --- | --- |
|  |  |
| Study code: | DurIRVac |
| EudraCT number: | 2021-000349-42 |
| Version number: | 1 |
| Date: | 2021-03-05 |
|  |  |
| Sponsor: | Sahlgrenska Academy, University of Gothenburg |
|  | |
| Principal Investigator: | Martin Lagging, MD, PhD, Professor |
|  |  |
|  |  |

Table of contents

[Signature page 5](#_Toc65495227)

[Contact information 8](#_Toc65495228)

[List of used acronyms and abbreviations 9](#_Toc65495229)

[1. Synopsis 10](#_Toc65495233)

[2. Background and rationale 12](#_Toc65495234)

[3. Benefit-risk evaluation 12](#_Toc65495235)

[4. Primary study objective 12](#_Toc65495236)

[4.1. Primary endpoint (variable) 12](#_Toc65495237)

[5. Study design and procedures 12](#_Toc65495238)

[5.1. Overall study design 12](#_Toc65495239)

[5.2. Procedures and flow chart 14](#_Toc65495240)

[5.3. Biological sampling procedures 15](#_Toc65495241)

[5.3.1. Handling, storage, and destruction of biological samples 15](#_Toc65495242)

[5.4. End of Study 15](#_Toc65495243)

[6. Subject selection 15](#_Toc65495244)

[6.1. Inclusion criteria 15](#_Toc65495245)

[6.2. Exclusion criteria 15](#_Toc65495247)

[6.3. Screening 16](#_Toc65495248)

[6.4. Withdrawal criteria 16](#_Toc65495249)

[7. Study treatments 16](#_Toc65495250)

[7.1. Description of investigational product(s) 16](#_Toc65495251)

[7.2. Randomization 16](#_Toc65495252)

[7.3. Blinding 16](#_Toc65495253)

[8. Methods for measurement of endpoints for clinical efficacy and safety 16](#_Toc65495254)

[8.1. Methods for measurement of endpoints for clinical efficacy 16](#_Toc65495255)

[8.2. Primary endpoint (variable) 17](#_Toc65495256)

[8.3. Methods for measurement of endpoints (variables) for clinical safety 17](#_Toc65495257)

[9. Handling of Adverse Events and Serious Adverse Events 17](#_Toc65495258)

[9.1. Suspected Unexpected Serious Adverse Reaction (SUSAR) 17](#_Toc65495259)

[9.2. Reporting of Suspected Unexpected Serious Adverse Reactions (SUSAR) 17](#_Toc65495261)

[10. Statistics 18](#_Toc65495262)

[11. Quality Control and Quality Assurance 18](#_Toc65495263)

[11.1. Quality Assurance and Sponsor oversight 18](#_Toc65495264)

[11.2. Monitoring 18](#_Toc65495265)

[11.3. Source data 19](#_Toc65495266)

[12. Ethics 19](#_Toc65495267)

[12.1. Compliance to the protocol, GCP and regulations 19](#_Toc65495268)

[12.2. Ethical review of the study and procedure for informed consent 19](#_Toc65495269)

[13. Collection, handling, and archiving data 21](#_Toc65495270)

[13.1. Case Report Form (Forskningspersonsformulär) 21](#_Toc65495271)

[14. Notification of study completion, reporting, and publication 21](#_Toc65495272)

[15. References 22](#_Toc65495273)

[16. Attachments 22](#_Toc65495274)

# Signature page

**Sponsor**

I am responsible for ensuring that this protocol includes all essential information to be able to conduct this study. I will submit the protocol and all other important study-related information to the responsible investigator(s) so that they can conduct the study correctly. I am aware that it is my responsibility to hold the staff members who work with this study informed and trained.

2021-03-05

| Sponsor’s/Principal Investigator’s signature Date  Martin Lagging, MD, PhD, Professor |
| --- |

Printed name

**Coordinating Investigator / Principal Investigator**

I have read this protocol and agree that it includes all essential information to be able to conduct the study. By signing my name below, I agree to conduct the study in compliance with this protocol, the Declaration of Helsinki, ICH GCP (Good Clinical Practice) guidelines and the current national and international regulations governing the conduct of this clinical trial.

I will submit this protocol and all other important study-related information to the staff members and investigators who participate in this study, so that they can conduct the study correctly. I am aware of my responsibility to continuously keep the staff members and investigators who work with this study informed and trained.

| I am aware that quality control of this study will be performed in the form of monitoring, audit, and possibly inspection. |
| --- |
| Coordinating Investigator/Principal Investigator’s signature Date  Martin Lagging, MD, PhD, Professor |

Printed name

**Principal Investigator**

I have read this protocol and agree that it includes all essential information to be able to conduct the study. By signing my name below, I agree to conduct the study in compliance with this protocol, the Declaration of Helsinki, ICH GCP (Good Clinical Practice) guidelines and the current national and international regulations governing the conduct of this clinical trial.

I will submit this protocol and all other important study-related information to the staff members and investigators who participate in this study, so that they can conduct the study correctly. I am aware of my responsibility to continuously keep the staff members and investigators who work with this study informed and trained.

I am aware that quality control of this study will be performed in the form of monitoring, audit, and possibly inspection.

|  |
| --- |
| Principal Investigator’s signature Date  Martin Lagging, MD, PhD, Professor |

Printed name

# Contact information

| **Role** |  |
| --- | --- |
| Sponsor | Martin Lagging, MD, PhD, Professor  Sahlgrenska Academy, University of Gothenburg  Guldhedsgatan 10B, Gothenburg, SE-413 46  Sweden  +46-(0)31-342 47 31  martin.lagging@medfak.gu.se |
| *Coordinating Investigator/ Principal Investigator* | Martin Lagging, MD, PhD, Professor  Sahlgrenska Academy, University of Gothenburg  Guldhedsgatan 10B, Gothenburg, SE-413 46  Sweden  +46-(0)31-342 47 31  martin.lagging@medfak.gu.se |
| *Clinical monitoring organization* | Gothia Forum  Guldhedsgatan 10C 413 46 Göteborg **+46-(**0)31 - 342 96 70 **E-post:** [gothia.forum@vgregion.se](mailto:gothia.forum@vgregion.se) |
|  |  |

# List of used acronyms and abbreviations

| **Abbreviation** | **Term/Explanation** |
| --- | --- |
| AE | Adverse Event = any untoward medical occurrence |
| AR | Adverse Reaction = adverse event, that is each unfavorable and unexpected reaction to a study treatment, regardless of dose |
| CRF | Case Report Form |
| DSUR | Development Safety Update Report = annual safety report |
| GCP | Good Clinical Practice |
| ICH | International Council for Harmonization |
| IMP | Investigational medicinal product |
| ITT | Intention-to-treat = including all data from all subjects who have participated in the study |
| LVFS | Läkemedelsverkets författningssamling (in English: Swedish Medical Products Agency’s statutes) |
| PP | Per Protocol analysis = including only data from subjects who have completed the study completely in accordance with the protocol, with no deviations from the protocol |
| SAE | Serious Adverse Event = serious untoward medical occurrence. SAEs include all serious events independent of whether they have a suspected causal relationship to the IMP or not. |
| SmPC | Summary of Product Characteristics |
| SUSAR | Suspected Unexpected Serious Adverse Reaction |
|  |  |
|  |  |

#

1. Synopsis

| EudraCT number: 2021-000349-42 |
| --- |
| Title: Evaluation of the Durability of Immune Responses to SARS-CoV-2 Using Novel Rapid Spike Interferon-Gamma Release and Activation-Induced Marker Assays and Anti-Spike IgG Following COVID-19 Vaccination |
| Study code: DurIRVac |
| Short background/Rationale/Aim: Vaccination against COVID-19 has now commenced in Sweden, although the durability of immune responses following vaccination for COVID-19 remains unclear. |
| Primary objective: To evaluate the durability of immune responses following vaccination for COVID-19 among immunocompromised, cirrhotic, renal insufficient and healthy participants using novel rapid spike interferon-γ release and activation-induced marker assays and anti-spike IgG serology, aiming at identifying correlates of vaccine-induced protection. |
| Study design: Phase IV post marketing surveillance, observational prospective cohort study |
| Study population: Immunocompromised, cirrhotic, renal insufficient and healthy participants |
| Number of subjects: 500 |
| Inclusion criteria:  1. Participants must give written informed consent  2. Woman or man aged 18 years or older undergoing vaccination against COVID-19 in Region Västra Götaland in accordance with clinic routine and regional prioritization. |
| Exclusion criteria:  1. Inability to give written informed consent  2. Inability to undergo blood sampling, for example lack of suitable blood vessels for sampling |
| Investigational product(s): The COVID-19 vaccines currently approved by EMA and in use in Region Västra Götaland:   1. Comirnaty, EMEA/H/C/005735, BioNTech Manufacturing GmbH 2. COVID-19 Vaccine Moderna, EMEA/H/C/005791, Moderna Biotech Spain, S.L. 3. COVID-19 Vaccine AstraZeneca, EMEA/H/C/005675, AstraZeneca AB. |
| Primary endpoint:  Levels achieved immediately before the 1^st^ and 2^nd^ doses, 4 weeks post-2^nd^ dose, and then every 3 months for 2 years for the following analyses:   1. anti-RBD Spike IgG quantitative serology calibrated against a WHO standard (Abbott) 2. a novel rapid spike interferon-γ release assay 3. a novel rapid spike activation-induced marker assay |
| Study period:  Q1 2021 – Q3 2023 |
| End of trial:  Q4 2023 |

# Background and rationale

Vaccination against COVID-19 has now commenced in Sweden, although the durability of immune responses following vaccination for COVID-19 remains unclear. The primary aim is to evaluate the durability of immune responses following vaccination for COVID-19 among immunocompromised, cirrhotic, renal insufficient and healthy participants using novel rapid spike interferon-γ release and activation-induced marker assays and anti-spike IgG serology, aiming at identifying correlates of vaccine-induced protection. Secondary aims include evaluating the impact of prior natural exposure to SARS-CoV-2 and human genetic variants on these vaccine responses, monitoring for emergence of vaccine-escape SARS-CoV-2 variants, and creating a large easily accessible biobank for research. To complement the data available through the Swedish National Vaccination Register, participants will enter detailed health-related information pertinent to the study in the case report form (CRF). The study will strive to sample participants immediately before the 1^st^ and 2^nd^ doses, 4 weeks post-2^nd^ dose, and then every 3 months for at least 2 years. Enrollment of participants at timepoints after initiation of vaccination will also be permitted, as sampling prior to vaccination may prove challenging.

# Benefit-risk evaluation

**Benefit:** By participating in the study, the participants can relatively rapidly find out if they have antibodies to SARS-CoV-2 via Direkttest.se if the sampling was registered there, which is the current clinical routine. The participants also contribute to development and furthering of knowledge with regards to COVID-19.

**Risk:** The participant will provide an extra serum tube (9 mL), three lithium-heparin tubes without gel (24 mL) and a saliva sample at each sampling other than that included in routine clinical practice. The discomfort of sampling and the invasion of integrity is considered minor. All statistical processing, compilation and reporting of the material takes place with the help of a decoded database in which the participants' identities remain unknown.

The scientific value of the study is judged to be great. The discomfort of sampling and the invasion of integrity is considered minor. Participants can relatively easily decide for themselves and thus make an informed decision about possible participation.

# Primary study objective

The primary objective of this study is to evaluate the durability of immune responses following vaccination for COVID-19.

## Primary endpoint (variable)

Primary variable: Levels achieved immediately before the 1^st^ and 2^nd^ doses, 4 weeks post-2^nd^ dose, and then every 3 months for at least 2 years for the following analyses:

1. a novel rapid spike interferon-γ release assay
2. a novel rapid spike activation-induced marker assay
3. anti-RBD Spike IgG serology (Abbott)

# Study design and procedures

## Overall study design

This study is an open phase IV post marketing surveillance, observational prospective cohort study. The researchers involved in this study have no influence or input with regards to which COVID-19 vaccine the participants receive, the number of vaccine doses, nor the timepoint of vaccination. The vaccination will be performed by healthcare providers in Region Västra Götaland in accordance with national and regional prioritization as well as the clinic routine.

Investigational products are the COVID-19 vaccines currently approved by EMA and in use in Region Västra Götaland, i.e.:

1. Comirnaty, EMEA/H/C/005735, BioNTech Manufacturing GmbH
2. COVID-19 Vaccine Moderna, EMEA/H/C/005791, Moderna Biotech Spain, S.L.
3. COVID-19 Vaccine AstraZeneca, EMEA/H/C/005675, AstraZeneca AB.

Region Västra Götaland, unlike any other region in Sweden, has negotiated access via Data&Analys to data from the Swedish National Vaccination Register for Covid-19 at an individual vaccinee level rather than on a population-based level. This data consists of (1) Date of vaccination, (2) Which vaccine was used, (3) Lot number, (4) Dose number, and (5) Healthcare provider that performed the vaccination. Currently it remains unclear whether information regarding possible redosing of vaccine will be available from the National Vaccination Register.

After obtaining informed consent, participants will be sampled (1 serum tube (9 mL), 3 litium-heparin tubes without gel (24 mL) and saliva) immediately before the 1^st^ and 2^nd^ doses (if this is not possible logistically, these samples may be obtained during the recommended 15-30 minute observation period after COVID vaccination), 4 weeks post-2^nd^ dose, and then every 3 months for a total of 2 years. Enrollment of participants at timepoints after initiation of vaccination will also be permitted, as sampling prior to vaccination may prove challenging. Sampling will be registered using Direkttest.se as currently is the norm.

The following analyses will be performed on samples from all participants:

1. a novel rapid spike interferon-γ release assay will be performed in the laboratory of Associate Professor Anna Martner.
2. a novel rapid spike activation-induced marker assay will be performed in the laboratory of Associate Professor Anna Martner.
3. Anti-spike SARS-CoV-2 IgG serology using the Abbott Architect newly developed assay for the detection of IgG antibodies (SARS-CoV-2 IgG II Quant). The assay is a fully automated CMIA Assay (Chemiluminescent Microparticle Immunoassay) for the qualitative and quantitative (AU/mL) determination of IgG antibodies against SARS-CoV-2, including the spike receptor-binding domain (RBD). This assay will be performed in the Department of Microbiology/Virology at Sahlgrenska University Hospital under the supervision of PI Professor Martin Lagging.
4. Directed single nucleotide polymorphism (SNP) genetic profiling of participants regarding *Interferon lambda-4* (*IFNL4*), *Inosine triphosphate pyrophosphatase* (*ITPA*), *NOX2*, *HLA-genotype, CD46, Interleukin 1,2,6,10 and12,* Blood group, *TLR3, UNC93B1, TICAM1, TBK1, IRF3, IRF7, IFNAR1, IFNAR2, RF7, IFNAR1*. These assays will be performed in the laboratory of PI Professor Martin Lagging.
5. Continuous monitoring for virological evidence of breakthrough post-vaccination SARS-CoV-2 infection via data obtained from the laboratory information system (LIS) at the Department of Clinical Microbiology/Virology, Gothenburg. These vaccine-breakthrough viruses will be sequenced (in particular regarding the spike protein) for evidence of vaccine escape mutants. Possible viral breakthrough variants will also be cultured in the BSL-3 laboratory and evaluated regarding sensitivity to neutralizing antibodies following vaccination and natural exposure.

The following analyses will be performed on samples from selected participants (e.g. those belonging to risk groups or other relevant subsets having received the various vaccines administered):

1. More extensive T-cell analyses evaluating possible immunologic memory after vaccination in the laboratory of Associate Professor Anna Martner.
2. Neutralizing antibody titration will be performed in the Biosafety Level 3 (BSL-3) laboratory at the Department of Microbiology/Virology at Sahlgrenska University Hospital. These analyses will be performed under the supervision of Associate Professor Kristina Nyström.
3. Genome-wide single nucleotide polymorphism sequencing for genome-wide association study (GWAS) using Illumina Infinium SNP Genotyping Arrays at National Genomics Infrastructure (NGI Sweden), SciLifeLab

##

## Procedures and flow chart

**

## Biological sampling procedures

### Handling, storage, and destruction of biological samples

At each study visit the following sampling will occur: one serum tube (9 mL) for analysis of antibodies to SARS-CoV-2 and three gel-free lithium heparin tubes (24 mL) for analysis of interferon-gamma and other markers. The biological material will be coded with a code key. the study will have access to the biological material for one year after the project is completed so that all analyzes have time to be performed. Thereafter all samples will be stored at Biobank Väst (co-administered by Region Västra Götaland and Sahlgrenska Academy).

## End of Study

The end of trial will be on March 1, 2024 by which time all data and sampling will have been collected and completed for the study population.

# Subject selection

- 1. Inclusion criteria

To be included in the study, subjects must meet the following criteria:

- The subject has given written consent to participate in the study.
- The subject must be woman or man aged 18 years or older undergoing vaccination against COVID-19 in Region Västra Götaland in accordance with clinic routine and regional prioritization.

## Exclusion criteria

Subjects must not be included in the study if any of the following criteria are met:

- Inability to give written informed consent
- Inability to undergo blood sampling, for example lack of suitable blood vessels for sampling

## Screening

Suitable study participants will be recruited from patients attending clinics at the Sahlgrenska University Hospital as well as healthy personnel working at the Dept. of Microbiology and Infectious Diseases Clinic at Sahlgrenska University Hospital.

Subject eligibility (that subjects fulfill all inclusion criteria and do not meet any exclusion criteria) is established before inclusion, treatment, or randomization.

## Withdrawal criteria

- The study subject may choose to discontinue the study at any time
- If they choose to *discontinue* their participation in the study, they do not have to state why, nor will it affect their future care or treatment. Cancelation of participation is easily performed by contacting the person responsible for the study.

# Study treatments

## Description of investigational product(s)

The approved COVID-19 vaccines will be used according to the approved indication. The relevant Summary of Product Characteristics (SmPC) is being submitted with the application.

## Randomization

No randomization will be performed in this study as the researchers involved in this study have no influence or input with regards to which COVID-19 vaccine the participants receive, the number of vaccine doses, nor the timepoint of vaccination. The vaccination will be performed by healthcare providers in Region Västra Götaland in accordance with national and regional prioritization as well as the clinic routine.

Subjects are included consecutively as they are found to be eligible for inclusion in the study. If a subject discontinues their study participation, their subject code will not be reused, and the subject will not be allowed to re-enter the study again.

## Blinding

The study is open and not blinded.

# Methods for measurement of endpoints for clinical efficacy and safety

- 1. Methods for measurement of endpoints for clinical efficacy

Levels achieved using the methods below will be monitored immediately before the 1^st^ and 2^nd^ vaccine doses, 4 weeks post-2^nd^ vaccine dose, and then every 3 months for 2 years for the following analyses:

1. anti-RBD Spike IgG quantitative serology calibrated against a WHO standard (Abbott)
2. a novel rapid spike interferon-γ release assay
3. a novel rapid spike activation-induced marker assay
   1. Primary endpoint (variable)

The primary endpoints (variables) are the levels achieved using the abovementioned assays, which will be monitored immediately before the 1^st^ and 2^nd^ vaccine doses, 4 weeks post-2^nd^ vaccine dose, and then every 3 months for 2 years. This study is an open phase IV post marketing surveillance, observational prospective cohort study.

- 1. Methods for measurement of endpoints (variables) for clinical safety

The endpoint(s) (variables) for clinical safety will be the reported adverse events (AE) and serious adverse events (SAE). This analysis will be performed continuously during the study.

# Handling of Adverse Events and Serious Adverse Events

Adverse events (AE) and serious adverse events (SAE) will be handled according to established health care standard reporting system.

- 1. Suspected Unexpected Serious Adverse Reaction (SUSAR)

A Suspected Unexpected Serious Adverse Reaction (SUSAR) is as stated by the abbreviation a suspected unexpected serious adverse reaction. This infers that the event is likely related to the administered IMP but that the occurrence of the event is unexpected and the adverse events that are not included in the Investigator’s Brochure (IB) or SmPC.

If sponsor detects a SUSAR this should be reported. When reporting a SUSAR (in English) the EU-common form CIOMS should be used.

- 1. Reporting of Suspected Unexpected Serious Adverse Reactions (SUSAR)

Those SAE which comes to the knowledge of the sponsor and is assessed to be SUSAR should be reported via a [CIOMS form](https://cioms.ch/wp-content/uploads/2017/05/cioms-form1.pdf) to the European Medicines Agency (EMA) EudraVigilance database according to the specified time frames.

# Statistics

This study is an open phase IV post marketing surveillance, observational prospective cohort study to determine the sustainability of immune responses to SARS-CoV-2 following natural infection and vaccination. As there are currently very few preliminary data, it is difficult to provide a statistical basis for the study population. We intend to initially examine the immune response of approximately 100 healthy researchers, 100 researchers with cirrhosis, 100 researchers with impaired kidney function, 100 researchers who have undergone organ transplantation and 100 researchers with impaired immune systems for other reasons.

# Quality Control and Quality Assurance

Quality Control (monitoring) and Quality Assurance (auditing) will be performed by Gothia Forum, Guldhedsgatan 10C, 413 46 Göteborg

telephone **+46-(**0)31 - 342 96 70)
**E-post:** [gothia.forum@vgregion.se](mailto:gothia.forum@vgregion.se)

## Quality Assurance and Sponsor oversight

The quality assurance systems and methods deemed appropriate by Gothia Forum will be used to assure that the study will ensure and control the quality as well having oversight of the study’s quality. This applies for the communication plan, training of study personnel, working manuals, meetings, central/local monitoring, audits etc.

The participant will provide an extra serum tube (9 mL) and three lithium-heparin tubes without gel (24 mL) at each sampling other than that included in routine clinical practice. The discomfort of sampling and the invasion of integrity is considered minor. All statistical processing, compilation and reporting of the material takes place with the help of a decoded database in which the participants' identities remain unknown.

The scientific value of the study is judged to be great. The discomfort of sampling and the invasion of integrity is considered minor. Participants can relatively easily decide for themselves and thus make an informed decision about possible participation. Thus, the risk to subjects posed by participating in the study is considered low.

## Monitoring

The study will be monitored by an independent monitor (Gothia Forum) before the study begins, during the study conduct, and after the study has been completed, so as to ensure that the study is carried out according to the protocol and that data is collected, documented, and reported according to ICH-GCP and applicable ethical and regulatory requirements. Monitoring is performed as per the study’s monitoring plan and is intended to ensure that the subject’s rights, safety, and well-being are met as well as data in the Case Report Form (CRF) are complete, correct, and consistent with the source data.

## Source data

The investigator must keep source documents for each subject in the study. A document describing what has been classified as source data in the study should be included in the Investigator Site File (ISF). The investigator must ensure that all source documents are accessible for monitoring and other quality control activities.

Source data is defined before study start at each individual site. The CRF is defined as source data in cases where data is not registered elsewhere, e.g., inclusion and exclusion criteria.

# Ethics

## Compliance to the protocol, GCP and regulations

The study will be performed in compliance with the study protocol, the Declaration of Helsinki, ICH-GCP (Good Clinical Practice) guidelines and current national and international regulations governing this clinical trial. This is to ensure the safety and integrity of the study subjects as well as the quality of the data collected.

## Ethical review of the study and procedure for informed consent

The final study protocol for clinical trials must be approved, as a part of the application for a permit for clinical trials, by both the Swedish Ethical Review Authority (Etikprövningsmyndigheten) and the Swedish Medical Products Agency before the trial can be conducted. The final version of the informed consent form and other information provided to subjects, must be approved or given a written positive opinion by the Swedish Ethical Review Authority.

The principal investigator at each site shall ensure that the subject is given full and adequate oral and written information about the study, its purpose, any risks and benefits as well as inclusion and exclusion criteria. Subjects must also be informed that they are free to discontinue their participation in the study at any time without having to provide a reason. Subjects should be given the opportunity to ask questions and be allowed time to consider the provided information. If the person chooses to participate, both the subject and the investigator shall sign the informed consent form. A copy of the subject information as well as the informed consent form shall be provided to the subject. The subject’s signed and dated informed consent must be obtained before performing any study-specific activity in the study. Each subject who participated in the study will be identified by a subject number on a subject identification list. The subject agrees that monitors, auditors, and inspectors may have access to their medical records and other source data. If new information is added to the study, the subject has the right to reconsider whether he/she will continue their participation.

# Collection, handling, and archiving data

Subjects who participate in the study are coded with a specific study identification number. All subjects are registered in a subject identification list (subject enrolment and identification list) that connects the subject’s name and personal number with a study identification number.

All data will be registered, managed, and stored in a manner that enables correct reporting, interpretation, and verification. The complete Trial Master File, as well as source documents, will be archived for at least 2 years after the study is completed. Source data in the medical records system is stored and archived in accordance with the respective hospital regulations.

## Case Report Form (Forskningspersonsformulär)

A Case Report Form (CRF) is used for data collection. The investigator must ensure that the registered data is correct, complete, and that reporting takes place according to the timelines that have been predefined and agreed. The investigator signs the completed CRF. A copy of the completed CRF will be archived at the study site.

# Notification of study completion, reporting, and publication

The Swedish Medical Products Agency shall be informed of the study’s completion at latest 90 days after study end, through submission of a ”Declaration of End of Trial Notification” form.

Within one year after the study is completed, the results shall be analyzed, a clinical study report with individual data shall be prepared, and the study results shall also be reported in the EudraCT database.

# References

# Attachments
