# Supplementary material for: Impaired SARS-CoV-2-specific T-cell reactivity in patients with cirrhosis following mRNA COVID-19 vaccination
Source: JHEP Rep. 2022 Apr 27;4(7):100496. doi: 10.1016/j.jhepr.2022.100496 (PMC9045869; doi:10.1016/j.jhepr.2022.100496)
Supplement: Multimedia component 4 [file mmc4.pdf]

LÄKEMEDELSVERKET
SWEDISH MEDICAL PRODUCTS AGENCY

Beslut

Kliniska prövningar-Licenser
Maria Bäärnhielm/ek

Datum: 2021-03-11

Eu-nr **2021-000349-42**
Dnr 5.1-2021-11118

Sahlgrenska Academy, University of Gothenburg
Professor Martin Lagging
Department of Infectious Diseases/Virology
Institute of Biomedicine
Guldhedsgatan 10B
413 46 Göteborg

Tillstånd till klinisk läkemedelsprövning
Prövningstitel: Evaluation of How Long Immune Responses to SARS-CoV-2 Persist Following COVID-19 Vaccination
Protokollnummer DurIRVac-1, protokollversion 1, 2021-03-05

Ni har ansökt om tillstånd att genomföra en klinisk läkemedelsprövning.

Läkemedelsverket lämnar med stöd av 7 kap. 9 § läkemedelslagen (2015:315) tillstånd att genomföra den kliniska läkemedelsprövningen.

Enligt LVFS 2011:19 ska ni skicka beslut från Etikprövningsmyndigheten till Läkemedelsverket senast 15 dagar från den dag då ni fick beslutet. Vänligen bortse från denna påminnelse om ni redan har skickat beslutet.

Detta beslut har fattats av gruppchef Lena Eriksson efter föredragning av klinikutredare Maria Bäärnhielm.

På Läkemedelsverkets vägnar

Lena Eriksson

Denna beslutshandling är inte underskriven. Detta påverkar inte beslutets giltighet.
