# Supplementary material for: Impaired SARS-CoV-2-specific T-cell reactivity in patients with cirrhosis following mRNA COVID-19 vaccination
Source: JHEP Rep. 2022 Apr 27;4(7):100496. doi: 10.1016/j.jhepr.2022.100496 (PMC9045869; doi:10.1016/j.jhepr.2022.100496)
Supplement: Multimedia component 5 [file mmc5.docx]

**JHEP Reports**

**CTAT methods**

Tables for a “Complete, Transparent, Accurate and Timely account” (CTAT) are now mandatory for all revised submissions. The aim is to enhance the reproducibility of methods.

- Only include the parts relevant to your study
- Refer to the CTAT in the main text as ‘Supplementary CTAT Table’
- Do not add subheadings
- Add as many rows as needed to include all information
- Only include one item per row

**If the CTAT form is not relevant to your study, please outline the reasons why:**

|  |
| --- |

- 1. **Antibodies**

| **Name** | **Citation** | **Supplier** | **Cat no.** | **Clone no.** |
| --- | --- | --- | --- | --- |
|  |  |  |  |  |

- 1. **Cell lines**

| **Name** | **Citation** | **Supplier** | **Cat no.** | **Passage no.** | **Authentication test method** |
| --- | --- | --- | --- | --- | --- |
|  |  |  |  |  |  |

- 1. **Organisms**

| **Name** | **Citation** | **Supplier** | **Strain** | **Sex** | **Age** | **Overall n number** |
| --- | --- | --- | --- | --- | --- | --- |
|  |  |  |  |  |  |  |

- 1. **Sequence based reagents**

| **Name** | **Sequence** | **Supplier** |
| --- | --- | --- |
|  |  |  |

- 1. **Biological samples**

| **Description** | **Source** | **Identifier** |
| --- | --- | --- |
|  |  |  |

- 1. **Deposited data**

| **Name of repository** | **Identifier** | **Link** |
| --- | --- | --- |
|  |  |  |

- 1. **Software**

| **Software name** | **Manufacturer** | **Version** |
| --- | --- | --- |
|  |  |  |

- 1. **Other (*e.g*. drugs, proteins, vectors etc.)**

| BNT162b2, Comirnaty, Pfizer-BioNTech |  |  |
| --- | --- | --- |
| mRNA-1273, Spikevax, Moderna |  |  |

- 1. **Please provide the details of the corresponding methods author for the manuscript:**

| Martin Lagging, Department of Infectious Diseases/Virology, Sahlgrenska University Hospital and University of Gothenburg, 413 45 Gothenburg, Sweden  [martin.lagging@gu.se](mailto:martin.lagging@gu.se) |
| --- |

**2.0 Please confirm for randomised controlled trials all versions of the clinical protocol are included in the submission. These will be published online as supplementary information.**

|  |
| --- |
